# Firearm Storage Behaviors — Behavioral Risk Factor Surveillance System, Eight States, 2021–2022

**DOI:** 10.15585/mmwr.mm7323a1

**Published:** 2024-06-13

**Authors:** Norah W. Friar, Molly Merrill-Francis, Elizabeth M. Parker, Carlos Siordia, Thomas R. Simon

**Affiliations:** 1 Division of Violence Prevention, National Center for Injury Prevention and Control, CDC.

**Summary**

**What is already known about this topic?**

Secure firearm storage is associated with lower risk for firearm injuries. Data on state and demographic variation in storage practices might help guide the development and implementation of prevention and evaluation efforts.

**What is added by this report?**

Among eight states with available data, 18.4%–50.6% of Behavioral Risk Factor Surveillance System respondents reported keeping a firearm in or around the home. Of respondents with a loaded firearm and a child or adolescent aged ≤17 years in the home, 25.2%–41.4% reported that a loaded firearm was kept unlocked.

**What are the implications for public health practice?**

State and demographic variation in storage practices highlights the importance of tailored prevention activities to reduce handling of guns by children and youths without adult supervision and other unauthorized persons.

## Abstract

Secure firearm storage might help reduce access by children and other unauthorized users and the related risk for injury or death. Information about state-specific prevalence of firearm storage practices can be used to develop secure storage messages and programs; however, such information is often unavailable. Data from the Behavioral Risk Factor Surveillance System, by respondent characteristics, were used to estimate prevalence of keeping firearms in or around the home and related storage practices for eight states that administered the firearm safety module in 2021 or 2022. Overall, 18.4% (California) to 50.6% (Alaska) of respondents reported that a firearm was kept in or around their home. Among those with a firearm in or around the home, 19.5% (Minnesota) to 43.8% (North Carolina) reported that a firearm was stored loaded. Across all eight states, approximately one half of those with a loaded firearm stored at least one loaded firearm unlocked. Among respondents with a child and a loaded firearm in the home, 25.2% (Ohio) to 41.4% (Alaska) reported that a loaded firearm was stored unlocked. Variability in firearm storage practices highlights the importance of local data and suggests opportunities to tailor prevention efforts to specific population groups to reduce risk for firearm handling by children without adult supervision, and other unauthorized persons. 

## Introduction

The firearm homicide rate in the United States declined slightly from 2021 (6.3 per 100,000 persons) to 2022 (5.9); however, the rate remained 34% higher than it was during 2019 (4.4) (*1*). The firearm suicide rate in 2022 (8.1 per 100,000 persons) increased since 2019, resulting in the highest rate since 1968 (the earliest year of data in CDC WONDER, an online public health database) (*2*). The presence of a firearm in the home has been associated with an increased risk for firearm homicide and suicide among household members, irrespective of their personal firearm ownership status (*3*). A risk for unintentional firearm injuries among youths also exists (*4*). These risks might be reduced by secure storage practices, including keeping a firearm unloaded and locked, especially among youths (*4*–*6*). Data on state- and population-specific storage practices are important for guiding the development and evaluation of tailored prevention activities. Data on adults’ reports of firearm storage practices were used to estimate the prevalence of firearms in or around the home and to examine related storage practices by sociodemographic characteristics.

## Methods

### Data Source

The Behavioral Risk Factor Surveillance System (BRFSS) is an annual, state-based, random-digit–dialed landline and mobile telephone survey that collects data on health-related behavioral risk factors and sociodemographic characteristics from noninstitutionalized adults aged ≥18 years in all 50 states, the District of Columbia, and participating territories. BRFSS includes core questions for all states, questions added by individual states, and optional modules, including the firearm safety module.* During 2021 and 2022, eight states administered the firearm safety module.^†^

### Statistical Methods

Weighted percentages and corresponding 95% CIs were estimated by state and stratified by whether the respondent indicated the presence of a child or adolescent aged ≤17 years living in the home and by sociodemographic characteristics, including sex, age group (18–24, 25–34, 35–64, and ≥65 years), and race and ethnicity (non-Hispanic Black or African American [Black], non-Hispanic White [White], non-Hispanic other [other], and Hispanic or Latino [Hispanic]). All respondents were included in the analysis, including those responding, “Don’t know,” “Not sure,” or “Refused” to any item in the firearm safety module. All analyses were conducted using SAS (version 9.4; SAS Institute) to account for survey design and complex weighting procedures. This activity was reviewed by CDC, deemed not research, and conducted consistent with applicable federal law and CDC policy.^§^

## Results

### Response Rate and Prevalence of Having a Firearm in or Around the Home

The mean combined response rate for BRFSS was 44.6% in 2021 and 45.9% in 2022. The percentage of respondents who declined to answer the first question of the module (i.e., whether any firearms are kept in or around the home) ranged from 3.6% (New Mexico) to 12.0% (Oklahoma). Among all respondents, the percentage of adults reporting a firearm kept in or around their home ranged from 18.4% (California) to 50.6% (Alaska) (Table 1).

**TABLE 1:** Proportion of persons reporting that a firearm is kept in or around their home, by state and respondent characteristics* — Behavioral Risk Factor Surveillance System, eight states,^†^ 2021–2022^§^

Characteristic	State and year of response, weighted % (95% CI)							
	Alaska 2021	California 2022	Minnesota 2022	Nevada 2022	New Mexico 2022	North Carolina 2021	Ohio 2022	Oklahoma 2021
**Firearm kept in or around home**								
Yes	50.58 (48.42–52.74)	18.42 (17.13–19.71)	37.12 (36.02–38.22)	35.63 (32.75–38.52)	35.87 (33.72–38.03)	37.43 (35.57–39.29)	37.75 (36.48–39.01)	38.99 (37.19–40.79)
No	38.39 (36.27–40.50)	75.73 (74.3–77.17)	57.41 (56.29–58.54)	58.08 (55.14–61.02)	60.27 (58.07–62.47)	51.38 (49.46–53.29)	55.02 (53.71–56.32)	48.11 (46.25–49.97)
Don’t know	—^¶^	0.66 (0.37–0.95)	0.37 (0.22–0.51)	—	—	1.19 (0.67–1.71)	0.58 (0.36–0.79)	0.92 (0.58–1.25)
Refused	10.26 (8.96–11.56)	5.19 (4.42–5.96)	5.10 (4.62–5.58)	6.01 (4.75–7.27)	3.64 (2.86–4.43)	10.00 (8.81–11.19)	6.66 (6.03–7.28)	11.99 (10.82–13.15)
**Respondents with firearm kept in or around home**								
**Sex**								
Female	48.11 (45.01–51.21)	14.62 (12.91–16.33)	31.15 (29.62–32.69)	32.56 (28.46–36.66)	29.26 (26.62–31.91)	33.52 (30.99–36.05)	32.72 (31.02–34.42)	35.57 (33.25–37.89)
Male	52.85 (49.82–55.88)	22.47 (20.54–24.40)	43.21 (41.66–44.77)	38.75 (34.65–42.85)	42.68 (39.33–46.03)	41.89 (39.16–44.63)	43.14 (41.26–45.02)	42.72 (39.94–45.49)
**Person aged ≤17 yrs in home**	53.48 (49.50–57.46)	18.40 (15.99–20.81)	37.61 (35.42–39.80)	35.74 (30.33–41.15)	38.71 (34.53–42.88)	36.21 (33.01–39.40)	40.56 (38.05–43.07)	38.92 (35.65–42.20)
**Age group, yrs**								
18–24	55.40 (47.21–63.60)	14.31 (10.77–17.85)	31.13 (27.07–35.18)	36.85 (25.73–47.97)	38.61 (30.07–47.14)	34.37 (26.83–41.91)	36.57 (31.88–41.26)	32.62 (26.05–39.19)
25–34	46.79 (41.02–52.56)	18.59 (15.33–21.85)	30.61 (27.52–33.69)	37.87 (30.01–45.73)	31.50 (25.17–37.84)	31.22 (26.63–35.81)	36.97 (33.35–40.58)	37.61 (32.46–42.77)
35–64	52.94 (50.02–55.86)	17.54 (15.72–19.36)	39.91 (38.39–41.44)	36.47 (32.31–40.64)	36.91 (33.88–39.95)	39.00 (36.43–41.57)	39.22 (37.43–41.02)	38.99 (36.51–41.46)
≥65	47.65 (44.05–51.25)	23.17 (20.21–26.12)	40.07 (38.11–42.03)	33.48 (28.82–38.14)	35.32 (32.06–38.59)	41.05 (37.43–44.66)	36.65 (34.56–38.74)	43.72 (40.74–46.71)
DK/NS/RA**	33.95 (21.9–45.99)	8.45 (3.71–13.18)	20.96 (13.88–28.03)	—	—	19.72 (10.29–29.15)	26.51 (17.32–35.69)	—
**Race and ethnicity^††^**								
Black or African American	34.45 (21.91–47.00)	21.99 (16.44–27.55)	17.36 (12.72–22.00)	27.39 (17.47–37.32)	29.23 (15.13–43.33)	30.20 (26.22–34.17)	29.63 (25.16–34.09)	24.88 (18.52–31.24)
White	55.01 (52.57–57.46)	25.51 (23.57–27.46)	41.98 (40.76–43.19)	44.59 (41.02–48.15)	42.84 (39.78–45.89)	44.51 (42.15–46.88)	40.08 (38.71–41.44)	43.32 (41.16–45.49)
Hispanic or Latino	51.79 (41.43–62.15)	13.57 (11.43–15.71)	15.76 (12.05–19.47)	19.93 (14.98–24.87)	32.46 (29.09–35.83)	11.42 (7.61– 15.22)	32.07 (24.06–40.08)	24.37 (18.28–30.46)
Other^§§^	42.60 (37.72–47.48)	15.11 (11.78–18.42)	24.77 (20.64–28.90)	42.78 (32.5–53.05)	27.67 (20.88–34.46)	27.52 (19.73–35.32)	27.28 (21.07–33.48)	37.21 (32.49–41.92)
DK/NS/RA^¶¶^	42.61 (32.08–53.15)	17.44 (12.09–22.79)	27.86 (21.98–33.74)	24.76 (11.52–37.99)	43.30 (31.18–55.43)	19.83 (9.11–30.55)	31.36 (23.41–39.30)	25.56 (13.69–37.42)

**Abbreviations:** DK = don’t know; NS = not specified; RA = refused to answer.

* Adults who reported a current firearm in or around their home.

^†^ Six states (Alaska, California, New Mexico, North Carolina, Ohio, and Oklahoma) administered the firearm safety module in 2021. In 2022, five states (California, Minnesota, Nevada, New Mexico, and Ohio) administered the firearm safety module. The most recent year of data is reported for each state.

^§^ Estimates are weighted to each state's adult population. Because denominators are among each sociodemographic characteristic, the prevalence estimates represent the behavior among a specific group.

^¶^ Dashes indicate data are not reported because the sample size is <30, or the absolute value of the CI is ≥0.30, or the relative CI width is >130% of the proportion.

** Respondents who reported “Don’t Know,” “Not Sure,” or “Refused” to the question about their age.

^††^ Persons of Hispanic or Latino (Hispanic) origin might be of any race but are categorized as Hispanic; all racial groups are non-Hispanic.

^§§^ Includes all American Indian or Alaska Native, Asian, Native Hawaiian or Pacific Islander, other race, and multiracial persons.

^¶¶^ Respondents who reported “Don’t Know,” “Not Sure,” “No Race Choice Given,” or “Refused” to the question about their race or ethnicity.

### Characteristics of Respondents with a Firearm in or Around the Home

The presence of a firearm varied by sociodemographic characteristics. For example, in all participating states other than Alaska and Nevada, a significantly higher percentage of men than women reported having firearms in the home (Table 1). Among White respondents, the presence of a firearm in the home ranged from 25.5% (California) to 55.0% (Alaska); among Black respondents, from 17.4% (Minnesota) to 34.5% (Alaska); among Hispanic respondents, from 11.4% (North Carolina) to 51.8% (Alaska); and among all other respondents, from 15.1% (California) to 42.8% (Nevada). The percentage of respondents with a child or adolescent aged ≤17 years in the home who reported having a firearm kept in or around the home ranged from 18.4% (California) to 53.5% (Alaska).

### Storage Characteristics of Firearms Kept in or Around the Home

Among respondents with a firearm kept in or around the home, 19.5% (Minnesota) to 43.8% (North Carolina) reported that a firearm was stored loaded (Table 2). Approximately one half of those who reported a loaded firearm reported that the loaded firearm was stored unlocked, ranging from 48.7% (Ohio) to 58.7% (Alaska) (Table 3). Among those who reported a loaded firearm and a child or adolescent aged ≤17 years in the home, 25.2% (Ohio) to 41.4% (Alaska) reported that a loaded firearm was stored unlocked. Across all states, approximately one half of respondents aged ≥65 years with a loaded firearm kept in or around their home reported that the loaded firearm was stored unlocked, ranging from 58.5% (New Mexico) to 72.5% (Oklahoma).

**TABLE 2:** Proportion of persons reporting having a firearm stored loaded among those reporting that a firearm is kept in or around their home, by state and respondent characteristic* — Behavioral Risk Factor Surveillance System, eight states,^†^ 2021–2022^§^

Characteristic	State and year of response, weighted % (95% CI)							
	Alaska 2021	California 2022	Minnesota 2022	Nevada 2022	New Mexico 2022	North Carolina 2021	Ohio 2022	Oklahoma 2021
**Storage of firearm in or around home**								
Loaded	29.70 (27.04–32.36)	25.69 (22.18–29.20)	19.46 (17.88–21.03)	39.24 (34.37–44.12)	40.09 (36.44–43.74)	43.82 (40.71–46.94)	37.06 (35.03–39.09)	41.20 (38.36–44.04)
Not loaded	66.47 (63.71–69.23)	69.9 (66.26–73.53)	77.51 (75.84–79.18)	57.21 (52.21–62.21)	57.44 (53.75–61.12)	52.74 (49.61–55.86)	59.89 (57.83–61.96)	54.06 (51.17–56.95)
Don’t know	3.15 (2.05–4.25)	3.71 (2.34–5.08)	2.53 (1.89–3.17)	3.44 (1.31–5.57)	2.43 (1.32–3.54)	2.66 (1.71–3.62)	2.52 (1.75–3.29)	3.68 (2.59–4.76)
Refused	0.68 (0.33–1.02)	—^¶^	—	—	—	0.78 (0.29–1.26)	0.53 (0.27–0.78)	1.06 (0.48–1.65)
**Firearm stored loaded**								
**Sex**								
Female	19.97 (16.77–23.17)	19.03 (13.69–24.37)	11.63 (9.63–13.64)	24.12 (18.37–29.88)	32.5 (27.55–37.45)	39.48 (34.88–44.09)	29.42 (26.66–32.18)	31.80 (28.16–35.45)
Male	37.82 (33.91–41.74)	30.32 (25.71–34.93)	25.22 (22.98–27.45)	52.13 (45.48–58.79)	45.44 (40.32–50.57)	47.79 (43.57–52.00)	43.28 (40.44–46.12)	49.73 (45.55–53.91)
**Person aged ≤17 yrs in home**	24.57 (20.06–29.07)	24.84 (18.37–31.32)	19.22 (16.11–22.33)	35.45 (26.65–44.24)	37.36 (30.74–43.99)	35.52 (30.02–41.01)	33.04 (29.33–36.75)	35.01 (29.91–40.12)
Age group, yrs**								
18–24	16.77 (9.85–23.68)	12.57 (4.97–20.17)	17.83 (11.53–24.12)	—	37.87 (23.89–51.84)	36.30 (23.34–49.26)	28.09 (21.12–35.07)	36.47 (24.82–48.11)
25–34	32.90 (25.16–40.64)	29.96 (19.37–40.56)	25.03 (19.49–30.58)	37.11 (24.2–50.03)	42.27 (30.15–54.38)	44.32 (35.58–53.06)	38.15 (32.3–43.99)	38.32 (29.76–46.89)
35–64	29.53 (25.94–33.11)	25.85 (21.02–30.69)	20.21 (18.1–22.32)	42.91 (35.82–50)	39.26 (34.37–44.16)	43.13 (38.95–47.31)	37.86 (35.03–40.68)	41.57 (37.68–45.45)
≥65	35.21 (30.25–40.18)	26.42 (19.84–33.00)	15.85 (13.41–18.28)	42.19 (34.26–50.12)	40.97 (35.26–46.68)	47.66 (41.81–53.51)	38.64 (35.13–42.14)	43.84 (39.35–48.32)
**Race and ethnicity^††^**								
Black or African American	—	42.82 (28.48–57.15)	31.71 (17.34–46.09)	—	—	42.77 (35.1–50.44)	50.72 (41.67–59.77)	40.93 (27.06–54.79)
White	31.06 (28.02–34.10)	26.97 (22.78–31.16)	18.21 (16.63–19.79)	42.67 (37.28–48.06)	43.88 (39.19–48.56)	44.57 (41–48.14)	34.63 (32.58–36.67)	40.91 (37.67–44.15)
Hispanic or Latino	35.98 (21.09–50.87)	18.53 (11.94–25.12)	27.63 (15.17–40.09)	37.03 (23.38–50.68)	37.65 (31.30–44.00)	28.44 (13.87–43.01)	29.61 (17.04–42.18)	40.02 (26.27–53.78)
Other^§§^	22.19 (16.46–27.92)	27.81 (16.2–39.43)	24.51 (16.25–32.76)	28.92 (14.57–43.27)	32.83 (19.3–46.36)	—	62.38 (50.03–74.73)	43.93 (36.45–51.40)
DK/NS/RA^ ¶¶^	—	24.22 (9.93-38.51)	30.22 (17.87–42.56)	—	—	—	36.93 (22.27–51.59)	—

**Abbreviations:** DK = don’t know; NS = not specified; RA = refused to answer.

* Adults who reported a current firearm in or around their home and load status.

^†^ Six states (Alaska, California, New Mexico, North Carolina, Ohio, and Oklahoma) administered the firearm safety module in 2021. In 2022, five states (California, Minnesota, Nevada, New Mexico, and Ohio) administered the firearm safety module. The most recent year of data is reported for each state.

^§^ Estimates are weighted to each state’s adult population. Because denominators are among each sociodemographic characteristic, the prevalence estimates represent the behavior among a specific group.

^¶^ Dashes indicate data are not reported because the sample size is <30, or the absolute value of the CI is ≥0.30, or the relative CI width is >130% of the proportion.

** Respondents who reported “Don’t Know,” “Not Sure,” or “Refused” to age were suppressed because the sample size is <30, or the absolute value of the CI is ≥0.30, or the relative CI width is >130% of the proportion.

^††^ Persons of Hispanic or Latino (Hispanic) origin might be of any race but are categorized as Hispanic; all racial groups are non-Hispanic.

^§§^ Includes all American Indian or Alaska Native, Asian, Native Hawaiian or Pacific Islander, other race, and multiracial persons.

^¶¶^ Respondents who reported “Don’t Know,” “Not Sure,” “No Race Choice Given,” or “Refused” to the question about their race or ethnicity.

**TABLE 3:** Proportion of persons reporting a loaded and unlocked firearm among those with at least one loaded firearm kept in or around the home,* by state and respondent characteristics — Behavioral Risk Factor Surveillance System, eight states,^†^ 2021–2022^§^

Characteristic	State and year of response, weighted % (95% CI)							
	Alaska 2021	California 2022	Minnesota 2022	Nevada 2022	New Mexico 2022	North Carolina 2021	Ohio 2022	Oklahoma 2021
**Locked status of loaded firearm kept in or around the home** ^¶^								
Unlocked	58.70 (53.38–64.03)	50.11 (42.06–58.17)	54.69 (50.16–59.23)	52.53 (44.94–60.13)	51.5 (45.53–57.47)	52.53 (47.73–57.34)	48.75 (45.34–52.16)	57.22 (52.8–61.65)
Locked	40.48 (35.15–45.82)	49.16 (41.13–57.18)	44.58 (40.06–49.10)	47.21 (39.61–54.80)	48.47 (42.5–54.44)	46.62 (41.82–51.42)	50.22 (46.81–53.64)	41.24 (36.83–45.64)
**Respondents with firearm loaded and unlocked**								
**Sex**								
Female	58.46 (49.66–67.25)	—**	44.33 (35.00–53.67)	42.59 (30.63–54.55)	45.6 (36.19–55.01)	51.76 (44.07–59.46)	45.7 (40.28–51.13)	45.04 (38.28–51.81)
Male	58.81 (52.15–65.48)	50.45 (41.20–59.70)	58.21 (53.05–63.38)	56.46 (47.18–65.73)	54.48 (46.85–62.11)	53.12 (46.95–59.28)	50.44 (46.06–54.82)	64.29 (58.59–69.99)
**Person aged ≤17 yrs in home**	41.42 (31.36–51.48)	—	38.95 (29.67–48.24)	30.58 (16.89–44.28)	38.04 (26.91–49.16)	37.20 (27.09–47.31)	25.24 (19.94–30.54)	37.02 (28.26–45.77)
**Age group, yrs** ^¶^								
18–24	—	—	—	—	—	—	42.42 (28.54–56.3)	—
25–34	54.51 (39.62–69.39)	—	51.19 (38.25–64.12)	—	—	47.78 (33.92–61.64)	44.54 (35.02–54.06)	42.75 (28.46–57.05)
35–64	59.90 (52.74–67.06)	40.22 (29.29–51.16)	51.73 (45.79–57.67)	49.27 (38.67–59.87)	47.90 (39.86–55.94)	48.18 (41.71–54.64)	45.57 (40.88–50.25)	56.14 (50.05–62.22)
≥65	64.59 (54.69–74.49)	61.53 (48.3–74.77)	68.38 (60.69–76.07)	63.98 (52.54–75.42)	58.53 (49.08–67.98)	62.85 (54.61–71.08)	58.96 (52.96–64.95)	72.51 (66.69–78.34)

* Adults who reported a current loaded firearm in or around their home and locked status.

^†^ Six states (Alaska, California, New Mexico, North Carolina, Ohio, and Oklahoma) administered the firearm safety module in 2021. In 2022, five states (California, Minnesota, Nevada, New Mexico, and Ohio) administered the firearm safety module. The most recent year of data is reported for each state.

^§^ Estimates are weighted to each state's adult population. Because denominators vary among each sociodemographic characteristic, the prevalence estimates represent the behavior among a specific group. In addition, estimates by race and ethnicity are not reported because of data suppression requirements.

^¶^ Respondents who reported “Don’t Know,” “Not Sure,” or “Refused” to questions on locked status or age were suppressed because the sample size is <30, or the absolute value of the CI is ≥0.30, or the relative CI width is >130% of the proportion.

** Dashes indicate data are not reported because the sample size is <30, or the absolute value of the CI is ≥0.30, or the relative CI width is >130% of the proportion.

## Discussion

In the eight states participating in the 2021 or 2022 BRFSS firearm safety module, 18.4%–50.6% of respondents reported the presence of a firearm in or around their home, and 19.5%–43.8% of those with a firearm reported that at least one firearm was stored loaded. Across states and sociodemographic groups, the household presence and storage of firearms varied, highlighting the importance of focused and culturally tailored efforts to enhance secure storage. For example, in at least 25% of homes in which the respondent reported having a child or adolescent aged ≤17 years in the home and a loaded firearm, at least one loaded firearm was stored unlocked. Previous research has demonstrated that most fatal unintentional firearm deaths among children and adolescents aged 1–17 years occur in a house or apartment, and that the firearms used were often stored loaded and unlocked and were discharged during play or when showing the firearm to someone else (*7*). These findings underscore the importance of discussing secure firearm storage practices with parents and caregivers, including supporting them in asking about the presence of unsecured firearms in other homes where their children visit and play, such as the homes of older family members.

These findings suggest an opportunity to examine factors associated with firearm storage patterns to improve secure storage messages and initiatives. Few studies have examined the effectiveness of prevention efforts in increasing secure storage, and most of those that do focus on health care providers during health care visits (*6*). However, a national survey of firearm owners found fewer than one in five (19%) selected physicians as “good” or “excellent” messengers to teach gun owners about secure storage practices, compared with approximately three quarters (77%) who selected law enforcement (*8*). In addition, few studies explore barriers and facilitators associated with secure storage. One national survey of firearm owners found that concern about home defense was selected by 43% of respondents as a factor influencing gun storage (*8*). Researchers have called for collaboration with diverse partners, including firearm owners, community members, and parents, to better understand the barriers and facilitators for focused and effective secure storage interventions (*9*).

Future research could evaluate community- and society-level approaches for increasing secure firearm storage and reducing firearm injuries. Providing secure storage devices (e.g., cable locks, trigger locks, and lock boxes) has been associated with improvement in firearm storage practices (*6*). Another approach implemented in some states is child access prevention negligent storage (CAP-NS) laws, which impose penalties on adults who allow children unsupervised access to unlocked firearms (*10*). Although reviews concluded that CAP-NS laws are associated with decreases in fatal and nonfatal firearm injuries in children (*5*), one recent study found that persons in states with CAP-NS laws were not significantly more likely to report storing guns locked than those who lived in states without these laws (*10*). Further, gun owners often did not know about their state’s CAP-NS laws (*10*). Additional research might increase understanding of how to effectively implement and raise awareness about existing laws among different sociodemographic groups and geographic regions, as well as the equity implications of these laws. Examples of ways to understand the equity implications include evaluating the implementation and enforcement of CAP-NS laws and determining the demographics of persons being prosecuted. Future research could continue to explore equitable strategies to increase secure firearm storage, including developing tailored messaging.

### Limitations

The findings in this report are subject to at least four limitations. First, wording of questions did not allow analysis of the locked status of firearms stored unloaded. Second, only eight states completed the firearm safety module in 2021 or 2022; thus, these findings might not be generalizable beyond these states. Third, a small percentage of respondents, 3.6% (New Mexico) to 12.0% (Oklahoma) declined to respond to the question asking whether they had a firearm in the home, and therefore did not complete the remainder of the firearm safety module, which might also affect generalizability of findings. Finally, BRFSS collects self-reported data, which is subject to social desirability and recall biases. Respondents might not feel comfortable disclosing the presence of a firearm in or around their home or storage practices, which could influence reported estimates.

### Implications for Public Health Practice

Researchers have suggested that secure storage practices might decrease the risk for firearm-related injuries and deaths among persons with a firearm in the home, particularly children and youths (*4*–*6*). Understanding the variation in state- and demographic-specific firearm storage behaviors might help state and local governments, community partners, and practitioners create focused approaches to decreasing firearm-related injuries and deaths in their communities. States administering the BRFSS firearm module have unique information that might be used to guide efforts within the state to increase secure storage practices and reduce potential for injuries associated with nonsecure firearm storage.

## Footnotes

* The prologue for the module instructs respondents to include firearms that are “kept in a garage, outdoor storage area, or motor vehicle.” The first question further instructs respondents to not include BB guns or guns that cannot fire. Respondents completing this module are asked, “Are any firearms now kept in or around your home?” Households of respondents who answer “Yes” are categorized as a household with a firearm, and respondents are then asked, “Are any of these firearms now loaded?” Respondents who report loaded firearms are asked, “Are any of these loaded firearms also unlocked?” The third question includes an explanation for unlocked: “By unlocked, we mean you do not need a key or a combination or a hand/fingerprint to get the gun or to fire it. Don’t count the safety as a lock.” Respondents who answer “No,” “Don’t know,” “Not sure,” or “Refused” at any point advance to the next module.

† Six states (Alaska, California, New Mexico, North Carolina, Ohio, and Oklahoma) administered the firearm safety module in 2021. In 2022, five states (California, Minnesota, Nevada, New Mexico, and Ohio) administered the firearm safety module. The most recent year of data is reported for each state.

§ 45 C.F.R. part 46.102(I)(2), 21 C.F.R. part 56; 42 U.S.C. Sect. 241 (d); 5 U.S.C. Sect. 552a; 44 U.S.C. Sect. 3501 et seq.
